# Coping and adjustment in men with prostate cancer: a systematic review of qualitative studies

**DOI:** 10.1007/s11764-017-0654-8

**Published:** 2017-10-23

**Authors:** Jason S. Spendelow, H. Eli Joubert, Haymond Lee, Bryony R. Fairhurst

**Affiliations:** School of Psychology, University of Surrey, Guildford, GU2 7XH UK

**Keywords:** Prostate cancer, Coping, Adjustment, Well-being, Systematic review

## Abstract

**Purpose:**

Prostate cancer (PCa) is one of the most common forms of cancer amongst males. Men’s coping responses are an important determinant of functioning and adjustment to this disease. Previous qualitative research exists in this area, but the current review sought to systematically review and summarise these studies.

**Methods:**

A systematic review was conducted to identify studies concerned with men’s coping strategies in their attempts to live with PCa. A search of relevant electronic databases was conducted to identify studies that met inclusion criteria for this review. Methodological quality assessment was also undertaken for each included study.

**Results:**

One hundred twenty-one publications were identified for initial screening, and 18 studies were included in the review. A total of five coping strategy categories or ‘meta-themes’ were identified across included studies. These categories were labelled ‘avoidance, minimisation, and withdrawal’, ‘directing cognition and attention’, ‘reframing masculinity and seeking support’, ‘retain pre-illness identity and lifestyle’, and ‘symptom/side-effect management’.

**Conclusions:**

A range of coping strategies were reported by men with PCa. Some of these strategies appear to be partially influenced by gender roles and masculinities. Coping meta-themes reported in this review have also been found in other research on men’s coping. Strategies relating to flexible interpretation of gender roles/masculinities may be a particularly relevant category of coping responses due to the hypothesised beneficial impact of flexibility on psychological well-being.

**Implications for cancer survivors:**

PCa survivors utilise a range of coping strategies, and the types of strategies used may have implications for men’s well-being. The ability to be flexible in both coping responses used, and in the view of oneself as a man may be particularly important skills in meeting the challenges associated with this disease.

## Background

Prostate cancer (PCa) is the most common form of cancer amongst males in the UK and is the second most common cancer amongst men in the USA [1, 2]. PCa tends to affect older men in that over 50% of those diagnosed are aged 70 and over [1]. As with other forms of cancer, PCa is associated with a range of symptoms and treatment side-effects including sexual dysfunction, urinary incontinence, bowel changes, pain, and fatigue [3]. These problems pose significant challenges to quality of life and well-being.

PCa is also associated with various adverse psychiatric, psychological, and quality of life outcomes. A meta-analysis by Watts and colleagues [4] found rates of depression and anxiety to be higher (across several PCa treatment phases) than those found in the general population. PCa is also associated with sub-clinical distress and reduced quality of life [5–7]. By contrast, some studies have reported relatively low rates of psychiatric disorder. Love et al. [8] found no significant differences in the prevalence of depressive disorders, and only slightly elevated levels of anxiety disorders in men with PCa compared with community controls. Relatively low levels of anxiety and depressive symptoms were similarly found by Bisson et al. [9]. Health outcomes for PCa are influenced by various physical and psychological factors, such as symptoms experienced [10], treatment received [11, 12], and extent of masculine identity threat [13]. There is also support for different mental health-related quality of life trajectories [14]. Combined with methodological differences, these factors may have contributed to variations in reported outcomes. Despite these variations, it is apparent that  significant numbers of men do indeed experience psychological distress, and this is perhaps the key message from the literature. Research that attempts to inform and alleviate such distress should be encouraged.

Given the adverse outcomes associated with PCa, coping patterns amongst men with this disease are of great importance. Coping is broadly conceptualised as a person’s attempts to manage stressful circumstances, along with the ascribed meaning or interpretation given to such circumstances [15]. Coping may be comprised of external (e.g. information seeking, engaging social support networks) and internal (e.g. humour, cognitive re-framing) strategies [16]. Link and colleagues [17] suggest that coping strategy selection in cancer is a multi-stage process incorporating a wide range of considerations, such as identification of potential coping strategies, goals of self and others, and illness severity.

There is an increasing body of qualitative research on the topic of coping in men with cancer. Wenger and Oliffe [18] indicated that, across various types of cancer, men use multiple coping strategies that can be categorised as ‘fortifying resources’ (knowledge building and physical strengthening), ‘maintaining the familiar’ (e.g. shield others from distress), or ‘getting through’ (e.g. restricting emotion). The use of multiple coping strategies has also been reported with PCa specifically [19] with Roesch et al. [20] making links between coping strategies and outcomes. Specifically, these authors suggested that approach (e.g. seeking information, acceptance, positive reappraisal), problem and emotion-focused (e.g. seeking practical support, humour) coping strategies were associated with positive psychological outcomes. Conversely, avoidance was related to negative adjustment. Other reviews in this area have been published [21, 22]. However, diffuse coping conceptual frameworks along with other methodological limitations (e.g. lack of detailed inclusion/exclusion criteria) have hindered such attempts to rigorously synthesise studies.

Various theoretical frameworks, including gender role socialisation, can be employed in understanding coping with a cancer context. This is because Courteney [23] argued that men’s health behaviour can be influenced by a need to ‘do’ masculinity. Connell’s theory of hegemonic masculinity [25] promoted the concept of pluralism in male gender socialisation (hence the term ‘masculinities’). Here, a particularly influential and idealised (hegemonic) form of masculinity is purported to exist that promotes a narrow, inflexible enactment of masculinity. Likely to be influenced by this are a set of masculine scripts in PCa that include ‘self-reliance’, ‘emotional control’, and ‘male sexual potency’ [24].

Despite the potential dominance of hegemonic forms of masculinity, it is said to exist amongst non-hegemonic (subjugated) forms of masculinity [25]. This raises the potential for more flexible interpretations of masculinity as documented in several recent studies. [26–28]. With illnesses such as PCa, coronary heart disease, and depression, some men re-negotiate masculinity in an attempt to cope with the medical and psychological consequences of physical health problems [29]. This research compliments work undertaken in the areas of psychological flexibility, positive masculinity, and coping flexibility, and the benefits such attributes confer on well-being [30–32].

Matheson et al. [33] proposed that adjustment in prostate cancer rests on the extent to which men can negotiate two key transitions labelled ‘gaining a sense of perspective over threats to testicular cancer’ and ‘striving to get on with life and restoring normality’. Successful adjustment in the first transition phase was said to involve strategies such as reflecting on positive meaning, positive reframing of cancer threat, active support seeking, and more flexible adherence to masculine values (amongst others). This last strategy reflects the practice of masculine role renegotiation as discussed above. The second transition was characterised by behaviours such as normalising bodily changes and resuming pre-illness occupational and social roles. There is some empirical evidence that these adjustment processes are adaptive. For example, acceptance and positive reframing have been associated with benefit finding in PCa [34].

### The current study

To summarise, PCa is serious disease with potentially wide-ranging implications for physical and psychological functioning. Coping and adjustment is a complex process, and research suggests that men’s strategies are wide-ranging and likely to be strongly influenced by masculinities. Empirical studies in this area have not been robustly summarised to date. However, in doing so, we may advance our understanding of how to support men affected by this illness.

The aim of the current review was to conduct a qualitative meta-summary [35] to identify self-initiated coping strategies reported by men diagnosed with PCa. In line with the above discussion, coping in the current review was operationally defined as any internal or external psychological strategy intended to manage stress regarding a diagnosis of cancer, the interpretation or meaning associated with diagnosis, and difficulties directly related to this medical condition (e.g. symptoms, treatment side-effects). The current review focused on qualitative studies. One advantage, amongst others, of primary qualitative research is that coping data are not influenced by a pre-determined set of strategies as is found in various coping inventories. In advancing our understanding of coping in PCa and how to support men more effectively, it is important to systematically review and synthesise these studies. As this review involved the analysis of secondary data only, ethical approval was not sought.

## Methods

### Eligibility criteria

Studies investigating men’s self-initiated coping strategies for PCa were eligible for inclusion in the current review. Coping strategies via formal interventions (e.g. support groups, individual psychological therapy) were not eligible for inclusion. Strategies that were not primarily psychological were also not eligible (e.g. symptom management via dietary changes). Studies needed to be primary empirical papers that used a qualitative research design and data-analytic strategy that yielded thematic categories. No other restrictions in study design were imposed. Study samples had to be comprised of adult males over the age of 18 who had a diagnosis of PCa (even though final participants were most likely to be much older than this). Studies needed to specifically report at least one coping strategy, based on the definition of coping provided above. Strategies that involved direct attempts to treat cancer were not eligible. Studies exclusively recruiting sexual minority men were excluded in order to facilitate analysis with a relatively homogenous group.

### Search strategy

**Fig. 1:**
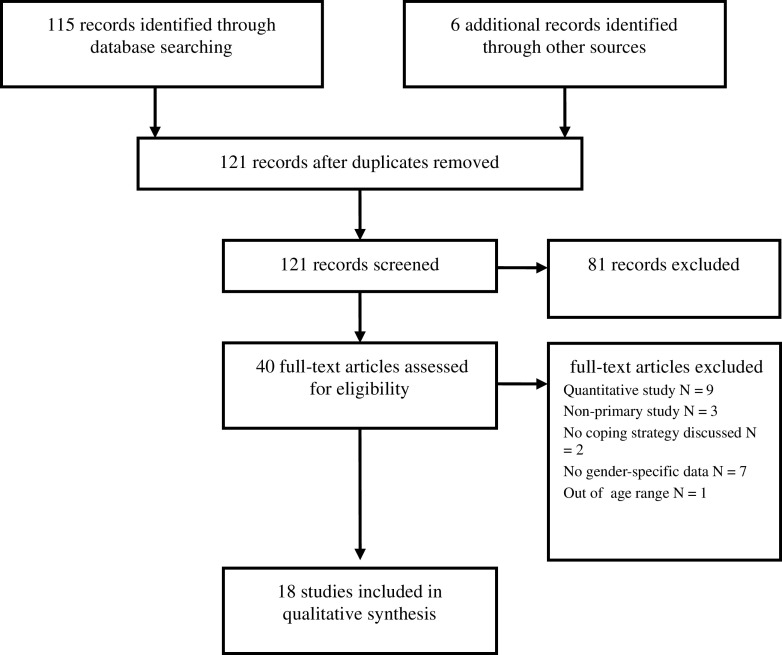
Flow chart of study screening process

Searches of the electronic databases PsychINFO and MEDLINE were conducted in April 2016 for English-language, published peer-review studies undertaken with adult male participants. No date restrictions were imposed. Search terms were developed from a preliminary search of existing studies in the area of coping and adjustment in informal male carers. Keywords were selected in the three concept areas of coping (“coping” OR “adaptation” OR “resilience” OR “self-help” OR “adjustment” OR “management” OR “strategies”), gender (“men(s)” OR “male(s)” OR “masculin*”) and cancer (“cancer” OR “neoplasms” OR “oncology” OR “tumour” OR “malignancy”). In addition, a hand search of reference sections within included studies was conducted to identify additional potentially relevant articles. A manual search of key journals was also conducted to identify additional articles. All publications returned from this search strategy underwent initial screening, followed by full-text review where eligible. See Fig. 1 for a summary of the study selection process.

### Data extraction and analysis

The approach to data extraction and analysis was the same as that adopted in previous research conducted in the area of men’s coping [26, 27]. Rationale and detailed description of this process have been previously provided so is not reproduced here. To briefly summarise, extraction and analysis took place in three stages:

1. Identification of relevant coping/adjustment text from individual study results sections. Coping in the current review was operationally defined according to Taylor and Stanton’s [16] definition as stated above.
2. Coding of text identified in stage 1 into broad meta-thematic coping domains, without restriction on number of domains generated. Seven domains were extracted at this stage for the current review.
3. Review and refinement of coping domains generated in stage 2. Categorisation of individual study themes into meta-themes was conducted by independent reviewers. These results were compared and disagreement resolved through discussion and consensus.

### Methodological quality assessment

Methodological quality assessment was undertaken for each included study. The Standard Quality Assessment Criteria for Evaluating Primary Research Papers from a Variety of Fields [36] was used for this purpose. This tool was used to assess each individual study in the domains of question/objective description, design identification and appropriateness, specification of study context, association with theoretical framework/body of knowledge, sampling strategy, data collection, data analysis, use of study credibility verification procedures, results–conclusions coherence, and reflexivity of potential impacts upon study data and findings. Individual studies were evaluated and assigned a score of zero, one or two for each of these 10 domains. A total quality score was then derived as listed in Table 1. Screening was conducted separately by two researchers (EJ and BF). Scoring results were compared and discrepancies resolved through discussion and use of a third scorer (JS) where necessary. The major methodological issue amongst the included studies was a lack of researcher reflexivity. There was no reflective account in seven of the included studies (four studies obtained a maximum score of two in this domain). Other issues involved inadequate/insufficiently detailed sampling strategies, theoretical frameworks, and study contexts.

**Table 1:** Study design, methodological quality, and reported themes

Study	Primary research aim	Data collection	Quality score (/20)	Thematic domain(s)
Appleton et al. (2015)	Investigate how men manage PCa and arriers/facilitators to disease adjustment	Purposive sampling Constructivist grounded theory Semi-structured interviews	16	• The diagnosis • The impact of prostate cancer and its treatment on daily life • Living with prostate cancer long term
Dieperink et al. (2013)	Investigate experience of treatment and rehabilitation including views on spousal involvement	Focus groups	12	• To cope with everyday life
Ervik and Asplund (2012)	Experience of bodily alterations during endocrine therapy	Semi-structured interviews Phenomenological hermeneutic approach	17	• Dealing with the alterations • To talk about cancer and the intimate details
Eziefula et al. (2013)	Investigate hot flush experiences	Semi-structured interviews	15	• Acceptance/adjustment • Management of flushes
Gannon et al. (2010)	Investigate how men construct and re-construct masculinity following radical prostatectomy	Semi-structured interviews Foucauldian Discourse Analysis	12	• Normalising impotence • Mental resilience
Hagen et al. (2007)	Investigate experience of PCa as context to explore new masculinities/ways of being	Semi-structured interviews Phenomenological analysis	18	• Fumbling in the dark • Threats to masculinity • In with the gang • The wise traveller
Hamilton et al. (2015)	Gain an understanding of how ADT impacts sexuality and how men cope	Social constructionism Semi-structured interviews	17	• Coping with sexuality concerns • Exercise to combat sexual health concerns
Hanly et al. (2014)	Investigate experiences of men with PC in sexual adjustment, informational, and support needs	Thematic analysis Semi-structured interviews	16	• Communication and support • Integration process
Hedestig et al. (2005)	Explore experiences of PCa and treatment	Content analysis Semi-structured interviews	13	• To bear the emotional experience of the illness alone • Striving for a sense of having control in a new life situation • Striving to become reconciled with a new life situation
Kazer et al. (2011)	Decision-making process for PCa treatment and coping	Thematic analysis Semi-structured interviews	13	• Going it alone • Sources of information and support • Coping strategies
Kelly (2009)	Masculine embodiment in PCa	Ethnographic analysis Semi-structured interviews and observational data	13	• Physical change: living with a new body • Restoring the masculine self • Seeing other men in the world
Levy and Cartwright (2015)	Strategies to promote and maintain well-being in PCa	Interpretative phenomenological analysis (IPA)	19	• Containing and revealing emotions • Striving towards the future • Living in the now • Taking care of the family
Maliski et al. (2008)	Identify processes used to maintain masculine identity with PCa	Grounded theory techniques	16	• Challenges to masculinity • Masculine identity renegotiation
McSorley et al. (2014)	Explore experiences and coping strategies to manage treatment side-effects	Thematic analysis	15	• Wife/partner support • Maintaining the routine • Positive attitude • Acceptance and stoicism • Living with symptoms
Nanton et al. (2009)	Experience of information and role in uncertainty management in PCa	Theme comparison/analysis	17	• Diagnosis • Reframing and problem solving following diagnosis • Treatment decisions • Living with uncertainty: adjustment and accommodation • Acceptance and understanding
Navon and Morag (2003)	Identification of coping strategies used by men during treatment	Thematic analysis (constant comparative method)	10	• Coping with bodily feminization • Coping with extinguished sexuality • Coping with constrained intimacy
Oliffe et al. (2009)	Identification of coping strategies to overcome uncertainty of active surveillance (AS) for PCa	Interpretive description	18	• The self-management of uncertainty in AS
Oster et al. (2013)	Describe experiences of men living with PCa	Content analysis	13	• Living with altered body experiences • Being empowered to live with the disease • Having support in everyday life

An initial list of six meta-thematic categories were generated, then reduced to five following refinement of category definitions. Each study was then reviewed by two independent coders (JS and HL) and relevant thematic content assigned under the applicable meta-thematic category. An individual study theme could be categorised within more than one meta-thematic category where appropriate. The categorisation of individual study themes was then compared between the two coders, and discrepancies resolved. The final categorisation of thematic content from the included studies can be found in Table 2.

**Table 2:** Meta-thematic categorisation of individual study themes

Study	Avoidance, minimisation, and withdrawal	Directing cognition and attention	Reframing and seeking support	Retain pre-illness identity and lifestyle	Symptom management
Appleton et al. (2015)	The diagnosis	The diagnosis The impact of prostate cancer and its treatment on daily life Living with prostate cancer long term	Living with prostate cancer long term		The impact of prostate cancer and its treatment on daily life Living with prostate cancer long term
Dieperink et al. (2013)		To cope with everyday life		To cope with everyday life	
Ervik and Asplund (2012)		Dealing with the alterations	To talk about cancer and the intimate details		
Eziefula et al. (2013)	Acceptance/adjustment Management of flushes				Management of flushes
Gannon et al. (2010)		Normalising impotence Mental resilience		Mental resilience	
Hagen et al. (2007)	Threats to masculinity	Threats to masculinity The wise traveller	In with the gang	The wise traveller	Threats to masculinity Fumbling in the dark
Hamilton et al. (2015)		Exercise to combat sexual health concerns Coping with sexuality concerns	Exercise to combat sexual health concerns	Exercise to combat sexual health concerns	Exercise to combat sexual health concerns
Hanly et al. (2014)	Integration process	Integration process	Communication and support		Integration process
Hedestig et al. (2005)	To bear the emotional experience of the illness alone Striving to become reconciled with a new life situation	Striving to become reconciled with a new life situation	Striving for a sense of having control in a new life situation	To bear the emotional experience of the illness alone Striving for a sense of having control in a new life situation	Striving for a sense of having control in a new life situation Striving to become reconciled with a new life situation
Kazer et al. (2011)		Coping strategies	Sources of information and support	Going it alone	Sources of information and support
Kelly (2009)		Physical change: living with a new body	Seeing other men in the world	Physical change: living with a new body Restoring the masculine self	Physical change: living with a new body Restoring the masculine self
Levy and Cartwright (2015)	Containing and revealing emotions	Taking care of the family Renegotiating purpose Living in the now	Containing and revealing emotions Striving towards the future	Containing and revealing emotions Taking care of the family Renegotiating purpose Striving towards the future	Taking care of the family
McSorley et al. (2014)	Maintaining the routine	Positive attitude Living with symptoms	Wife/partner support	Maintaining the routine Acceptance and stoicism	Living with symptoms
Maliski et al. (2008)	Challenges to masculine identity	Masculine identity renegotiation	Masculine identity renegotiation	Challenges to masculine identity	
Nanton et al. (2009)		Reframing and problem solving following diagnosis Living with uncertainty: adjustment and accommodation Acceptance and understanding	Living with uncertainty: adjustment and accommodation		Reframing and problem solving following diagnosis Treatment decisions Living with uncertainty: adjustment and accommodation
Navon and Morag (2003)		Coping with extinguished sexuality Coping with constrained intimacy		Coping with constrained intimacy	Coping with extinguished sexuality Coping with bodily feminization
Oliffe et al. (2009)	The self-management of uncertainty in AS		The self-management of uncertainty in AS	The self-management of uncertainty in AS	The self-management of uncertainty in AS
Oster et al. (2013)	Having support in everyday life	Being empowered to live with the disease	Having support in everyday life	Being empowered to live with the disease	Living with altered body experiences

## Results

### Description of included studies

In total, 18 studies meet inclusion criteria for the qualitative synthesis. See Fig. 1 for a flow chart of the study screening protocol. Details for the included studies can also be found in Table 3. There was an overall sample size of 389 taken from studies published between 2003 and 2015. Data were drawn from several countries, most notably the UK (7). Many of the remaining studies originated from continental Europe and North America. The sample comprised mainly of middle and older-aged men with an age range of 45 to 85 (three studies did not provide age data). Other sociodemographic data were reported with little consistency. Nine studies indicated that proportion of their samples identifying as retired. This group comprised at least 50% of participants in six of these studies (range 33–100%). In the seven studies that reported relationship status data, the label of ‘married’ was used most frequently. Participants in this category comprised the majority of participants in all of these studies (range 66–90%). Overall, limited sociodemographic data were presented in these studies. This limited the extent to which the overall sample could be characterised.

**Table 3:** Details of included studies

Study	*N*	Age range	Country	Demographic information	Diagnosis/treatment details
Appleton et al. (2015)	27	57–76	United Kingdom (UK)	Retired 22 ‘Most’ lived with spouse	• Study participation prior to and up to 18 months after radiotherapy • Combination of surgery and/or hormone therapy in addition to radiotherapy
Dieperink et al. (2013)	13	66–77	Denmark	‘None came from an ethnic minority’	• Primary PCa (stages T1–T3) • Treated with radiotherapy and androgen deprivation therapy (ADT)
Ervik and Asplund (2012)	10	56–83	Norway	8 retired 9 married/‘female friend’	• Diagnosed within 4 years prior to study • Endocrine therapy (primary treatment) for localised/local advanced PCa
Eziefula et al. (2013)	19	45–84	UK	White 15, Black 4 Married/cohabitating 15 Retired/unemployed 14	• Any stage of PCa • Received ADT • Experiencing hot flushes
Gannon et al. (2010)	7	58–70	UK	Married/partner 5 Retired 5 British 4	• Treated for localised PCa by radical prostatectomy • Interviewed 7–15 months post-surgery
Hagen et al. (2007)	15	49–80	Canada	Retired 5 Married 13	• Diagnosed at least 6 months prior to study • Received ‘some form of treatment’
Hamilton et al. (2015)	18		Australia	Married 12 Heterosexual 17	• Received ADT for up to 12 months
Hanly et al. (2014)	21	50–69	Australia	Married 16 Heterosexual 20 Retired 7, full/part-time employed 13	• Diagnosed and treated for PCa in the 5 years prior to the study
Hedestig et al. (2005)	10	61–79	Sweden	Married 9 Retired 5	• Diagnosed with localised PCa • Finished external beam radiotherapy between 6 months and 3 years prior to the study
Kazer et al. (2011)	17	47–72	USA	Single 17 Caucasian 14	• Treatment received 5–8 years prior to study • Participants underwent external beam radiotherapy, radical prostatectomy surgery, and brachytherapy
Kelly (2009)	14	No data	UK	Unclear from information provided	• ‘Recently diagnosed’ with PCa
Levy and Cartwright (2015)	5	50–72	UK	Married/long-term relationship 5 Retired 5 White 5	• Diagnosed 3–10 years prior to study • Advanced PCa • Various treatments received
Maliski et al. (2008)	95	50–70+	USA	Latino 60 African American/Black 35	• Treatment received less than 12 months to over 2 years prior to the study • Various treatments received
McSorley et al. (2014)*	11	No data	UK	No data	–
Nanton et al. (2009)	36 22	55–85	UK	Phase 1: married 31(/40) Phase 2: married/cohabitating 14(/18)	• Diagnosed at least 4 weeks prior to study • Various treatments
Navon and Morag (2003)	15	57–85	Israel	Married 13 Retired 7	• Receiving hormonal therapy for 6 months-3 years plus various other treatments
Oliffe et al. (2009)	25	48–77	Canada	Married 19 White 19	• Asymptomatic men with low-risk, early-stage PCa
Oster et al. (2013)	9	No data	Sweden	No data	• Receiving radiotherapy

Study design information is summarised in Table 1. Semi-structured interview was the most frequently used data collection method. Focus groups were used in only one study [37]. A range of data analytic procedures were employed across included studies, notably thematic analysis (or some variation). Aims of individual studies broadly corresponded with topics related to coping and adjustment. Most studies focused on adjustment to PCa symptoms and treatment side-effects, along with implication of PCa for identity and masculinities.

### Coping and adjustment meta-themes

Following analysis of data from the individual included studies, a total of five meta-themes were derived under which study-level thematic domains could be classified. Meta-thematic categories were ‘avoidance, minimisation, and withdrawal’, ‘directing cognition and attention’, ‘reframing and seeking support’, ‘retain pre-illness identity and lifestyle’, and ‘symptom management’.

#### Avoidance, minimisation, and withdrawal

This meta-thematic category comprised coping strategies intended to prevent engagement with PCa in terms of medical issues experienced, and discussions with others about the disease. This group of strategies involved active attempts to avoid psychologically confronting PCa and its consequences. In some studies, general avoidance strategies were reported, involving distraction [38, 39] and alcohol [40]. Avoidance of the illness itself involved non-engagement with information seeking and receipt. For example, some participants in Hanly et al. [40] reported not reading patient information materials. In some studies, participants talked about minimisation of side-effects and ‘not wanting to make a big deal’ of their medical circumstances [41]. There was also a degree of avoidance or distancing from other people. In Hedestig et al. [38], participants reported active avoidance of social contact. This was discussed elsewhere and termed ‘withdrawing into themselves’ [42] The expression ‘emotional distancing’ was used in Levy and Cartwright [43], while some men reported ‘going it alone’ during treatment [44].

#### Directing cognition and attention

This category involved active attempts to orientate, direct, and/or reframe thoughts about PCa in a positive manner. Strategies in this area reflected an over-arching attempt to influence cognition and create a positive attitude to both present circumstances and the future. In terms of general attitudinal orientation, strategies such as ‘think positively’ [40, 43, 44] and ‘waiting hopefully’ [45] were reported without substantial elaboration. Humour was also used by participants in several studies [37, 42, 46–48].

Many strategies involved attempts to positively reframe how symptoms and side-effects were viewed. This primarily involved characterising PCa symptoms and side-effects as normal consequences of growing old, rather than exclusively part of an underlying illness [37, 42, 45, 49, 50]. For example, Gannon et al. [49] reported that participants viewed erectile dysfunction as ‘developmentally normative’. Perhaps as a consequence of side-effects, some men attempted to shift partner relationship priorities away from sexual contact and into other areas such as relationship quality. For instance, some men in Navon and Morag [51] emphasised spouse as a best friend and looked for non-sexual forms of intimacy. In another approach, participants sometimes managed treatment side-effects by viewing them as preferable to the consequences of no treatment [47, 51]. Others reported a focus on the benefits of treatment as a way to cope with side-effects [42]. Finally, some participants attempted to adopt a positive attitude towards prognosis, as seen in McSorley et al. [39]. For example, anticipating symptoms to be short term and/or likely to improve [50]. In other cases, participants focused on holding medical staff in positive regard and placing faith in their clinical competences [38, 44].

Beyond a reframing of symptoms and side-effects, attempts to be present or future orientated (rather than focused on the past) represented another cognitive strategy. This was labelled ‘optimistic perseverance’ in Levy and Cartwright [43]. This sometimes involved a focus on the past in order to draw upon positive lessons for future health and lifestyle. In Hagen et al. [47], participants reported attempts to identify positive illness lessons, and to challenge existing attitudes towards life. In Appleton et al. [42], participants were reported to have reflected on the past in order to identity how to have a more positive effect on health in the future. Attempting to focus on the present time was discussed in Hedestig et al. [38] and Levy and Cartwright [43], whereby participants attempted to be ‘present-focused’ in their everyday activities (e.g. recreational pursuits).

#### Reframing masculinity and seeking support

In this category, participants attempted to broaden and/or more flexibly interpret masculinities. Support seeking was subsumed within this meta-theme because reinterpretation was often a precursor to support seeking. Across several studies, participants questioned previous or traditional masculine roles and characteristics [45, 52]. This sometimes resulted in men reporting a changed or expanded sense of identity [45], or adopting a more flexible masculine identity [45]. Participants in Kelly [52] reported a changed form of masculinity which involved a new awareness of vulnerability. Alternatively, accepting weakness was reported in Oster et al. [53]. In Levy and Cartwright [43], some men paid more attention to striking a balance between expressing and controlling emotions. This involved a re-interpretation of bravery as being able to display emotion to others. In Maliski et al. [45], the attributes of ‘strength’ and ‘control’ became associated with behaviours such as eating better and going to medical appointments.

In several studies, participants placed increased emphasis on relationships and interpersonal skills [45, 50]. This was generally permissible only once existing masculine traits had been challenged. Men in several studies sought support and talked to others more often [42, 44], however, there was often restriction of communication to spouses, immediate family members, and/or those with direct knowledge of PCa [38–40, 46–48, 54]. Change in relationship dynamics with partners was a particular focus in some studies. In Maliski et al. [45] participants reported a shift in importance away from physical attributes to relational skills (e.g. placing more value on relationships, emphasising respect of women rather than dominance). De-emphasis of physical and sexual intimacy was also reported in Navon and Morag [51] and represents a response to physical changes that contrasts with avoidance (as discussed under the avoidance, minimisation, and withdrawal meta-theme).

Mutual support and comradery with other men diagnosed with PCa appeared to be a particularly important source of social support to some [53]. An opportunity to talk to other men in similar circumstances was valued [38]. Support extended to help in managing side-effects [50]. In one study, there was a desire to educate other men about PCa and support newly diagnosed men [47]. This idea of taking on new meaningful social roles was seen elsewhere in terms of taking on volunteering roles [43].

#### Retain pre-illness identity and lifestyle

Participants reported strategies that involved attempts to consolidate or return to pre-illness identity, behaviours, and social roles. Strategies in this category primarily reflected efforts to promote gender roles and characteristics that influenced life prior to PCa. Most commonly, this was pursued in an attempt to retain ‘former masculine identities’ [52]. This was mainly achieved  through attempts to retain or assume a ‘leader’ role [45] such as father or 'protector' [43]. Some emphasised the importance of continuing in employment (if not retired), and remained working where possible [52]. Attempts to exhibit emotional restraint were common. This occurred in responding to diagnosis [49] and was sometimes seen as a way to protect others from distress and fears [43, 45]. General stoicism in the face of diagnosis and/or bodily changes was reported in several additional studies [45, 47, 52].

Three further sub-themes were evident with this meta-theme. One was that of ‘taking control’ by actively managing aspects of the treatment process, such as controlling communication [43] and by making practical plans for the future (e.g. financial planning) [38]. Another involved actively exhibiting valued masculine traits through physical exercise or strengthening in response to bodily changes [50, 52, 53]. A third theme involved conceptualising PCa in mechanistic terms, such as seeing the illness as a problem that needed to be ‘fixed’ [47] or conceptualising treatment as [53].

#### Symptom/side-effect management

This meta-theme included participant strategies aimed to manage the physical impact of illness symptoms and treatment side-effects. The majority of coping approaches under this meta-theme involved behavioural strategies. For instance, adopting a healthier lifestyle was often reported, usually in terms of improved diet and increased exercise [40, 48]. Planning and problem-solving around treatment side-effect issues were also referred to in the included studies [38, 40, 41]. For example, men in Hanly et al. [40] talked about reducing fluid intake to cope with bladder control issues. Seeking general medical information about PCa was also common [43, 44, 47, 52]. Another strategy regarding side-effect management was physical concealment of side effects and one’s body because issues such as body shape change were of great concern to men [39, 45]. In one study, this also involved participants not looking in the mirror, with approaches such as these appearing to reflect a desire to appear ‘normal’ [51].

## Conclusions

The current review summarised a small but significant body of qualitative studies examining coping and adjustment of men diagnosed with PCa. A total of five meta-thematic categories were derived from this literature: avoidance, minimisation, and withdrawal; directing cognition and attention; reframing masculinity and seeking support; retain pre-illness identity and lifestyle; and symptom/side-effect management. While previous reviews of coping and adjustment have been conducted in this area [21, 55], the current review differs in its scope, inclusion criteria, and approach to data synthesis. The current review provides important and novel insights into the nature of coping and adjustment of men diagnosed with this disease. This review adds to similar reviews and draws important parallels with men’s coping in other domains [26, 27]. A gender socialisation framework was utilised to contextualise the selection and use of coping strategies. However, it is important to acknowledge that the coping strategies identified are not exclusive to males, and there are many other potential influences in the coping process. For example, avoidance and withdrawal may also result from underlying psychological issues such as anxiety and depression.

Avoidance, minimisation, and withdrawal relates to strategies aimed at the avoidance of engagement with PCa physically, psychologically, and interpersonally. Such strategies may reflect the dominant masculine scripts such as ‘self-reliance’ and ‘emotional control’ as detailed by Burns and Mahalik [24]. These may represent efforts to minimise impact of side-effects as threats to masculinity [47]. This approach to coping is likely to be undesirable given the potential link between restricted emotional expression and psychological adjustment [20, 56]. This category is comparable to meta-themes reported elsewhere with men’s coping in depression (‘social concealment and minimisation’) and men functioning in a carer role (‘promoting masculinities and taking charge’) [26, 27]. In other words, suppression and minimisation are coping approaches exhibited by some men across a diverse range of situations.

Directing cognition and attention was a meta-theme that involved men attempting to employ a positive focus and reframing of symptoms and treatment side-effects. Similar to men who ‘seek new perspectives’ as a way to cope with depression [26], directing attention appears to be a coping strategy whereby men draw out aspects of their circumstances for which they can draw optimism and gratitude. Englar-Carlson and Kiselica [31] have outlined a positive psychology/positive masculinity model in which they identify socialised male strengths in several areas. One such area is humour, which was a feature of this meta-thematic category. This suggests that, through gender socialisation, men may be able to utilise strategies such as humour more easily than other strategies. Directing cognition in a positive way may be an approach to be encouraged. According to Matheson et al.’s [33] theory, positive reframing is also a process associated with adaptive adjustment to testicular cancer.

The current review indicated that some men engage in a process of reframing and seeking support. In line with theoretical work suggesting the pluralistic nature of masculinity [25], along with masculine identity flexibility and re-negotiation [27, 28], this meta-thematic category suggests that some men cope with PCa through actively re-positioning their identity in non-hegemonic terms. Men also adopt a more flexible interpretation and enactment of masculinity. Such strategies are again found elsewhere. This process has been labelled ‘creating new behaviours, roles, and identities’ amongst male carers [27] and ‘promote flexible masculinity’ in men affected by depression [26]. It appears that there may be different trajectories of resistance to masculine norms [28], thereby explaining why some may adopt and others not adopt strategies within this meta-theme. This re-framing and flexibility may enable men to access greater levels of social support than that which was possible in those who strongly endorse hegemonic ideals. However, men are still highly selective around support seeking and generally limit this to partners, close family members, and others diagnosed with PCa. We could assume this meta-thematic category represents adaptive coping responses. For example, psychological flexibility is linked with positive adjustment [32].

Retain pre-illness identity and lifestyle is a meta-theme that encapsulates men’s efforts to maintain ‘normality’ or a familiar, pre-illness lifestyle. This approach has again been noted in male carers and men affected by depression [26, 27]. Men in the included studies appeared to approach this ‘status-quo’ objective largely through exhibiting traditional Western masculine characteristics. The diagnosis may be a context in which men attempt to ‘do masculinity’ [23]. Additionally, because some men with PCa report diminished masculinity [57], there may be an attempt by some to build masculine capital [58] given the perceived threats to masculinity that some men report [59]. The use of strategies within this meta-theme is of concern given the association of traditional masculine scripts with psychological maladjustment [60, 61].

Strategies reported under the meta-theme symptom/side-effect management appeared to be in contrast with the first meta-theme (avoidance, minimisation, and withdrawal) in that there were several approach- and problem-solving-orientated strategies reported. This meta-theme also represents responses to physical issues (e.g. treatment side-effects) rather than psychological ones (e.g. adjustment). While the presence of symptoms and side-effects on their own necessitate a coping response, it could be argued that some men are more comfortable in engaging with practical coping strategies. A focus on practical tasks was reported by carers in Spendelow et al. [27] where it was hypothesised that this might reflect a number of functions, such as using the body itself to fulfil social roles (e.g. a leader or initiator) as represented in Watson’s [62] male body schema and to avoid negative affect.

The current findings apply to older men, given the age range of study participants. It, therefore, cannot be assumed that coping strategies discussed here are applicable to men at other developmental stages and/or with other illnesses (including other forms of cancer). There also might be various contextual and social variables that influenced these findings (e.g. cohort effects) that may have had a unique influence on the current review findings. Furthermore, issues of sexuality are relevant given the focus of included studies on heterosexual men. The coping experiences of sexual minorities may be different and require separate examination. Having said this, there is a general lack of research on masculinities and older men. This is significant because issues such as retirement, social relationships, and caregiving represent specific issues which may have a substantial effect on the health of older men [63]. The current review has made a contribution to this lack of research with older male participants, in identifying that masculinities are likely to heavily influence coping in this age group.

### Clinical implications

Men with PCa adopt numerous strategies to cope and adjust to the many challenged raised by this illness. The findings of the current review suggest the possibility that gender socialisation and masculinities may contribute to the selection and use of these strategies. There is a clear contrast to approaches where men either attempt to conform with or re-shape/interpret masculine identities. There is some evidence that the latter approach is associated with more positive health outcomes. Thus, knowing the types of strategies men employ is important in guiding health professional efforts to support adaptive coping in men with PCa. Refinement of psychological and psychoeducational interventions is needed because, while existing interventions may have a therapeutic effect over and above routine care, such gains may not be sustained [64].

Because there is some evidence to suggest that flexibility in masculinities and coping is adaptive, it may be appropriate to focus on interventions that promote this skill. There are various ways in which this can be achieved. For instance, certain psychotherapeutic approaches incorporate psychological flexibility in the conceptualisation of mental health difficulties (e.g. Acceptance and Commitment Therapy (ACT) [65]). Alternatively, Way et al. [28] identified a number of social, cultural, and interpersonal factors that appear to facilitate resistance to masculine norms. One of these is having close relationships. Some men within studies included in the current review reported disclosure and social support seeking (reframing and seeking support). Helping men to strengthen and utilise social networks could be a way to increase flexibility provided these networks embody characteristics congruent with flexibility (e.g. empathy, interdependence). Support-seeking is further important because men and their partners have a reciprocal influence to the diagnosis of PCa [66], the illness has an effect on partner well-being [67], and married men with high partner support reported lower levels of distress than unmarried men or those married with low support [68].

### Future research

There are a number of important avenues for future research highlighted by the findings of this review. Firstly, the current body of studies represent a fairly homogenous group of men, and the literature would benefit from extending these findings to more diverse samples. For example, exploring the impact of sexuality, social class, and cultural and ethnic background might influence the selection of coping strategies chosen by men in response to PCa. This would assist us to understand whether the patterns of coping identified in this review are generally utilised across diverse groups of males with PCa, or represent styles of coping used by a specific group (and influenced by socio-cultural variables, etc.). Additionally, while the studies included in this review help us to better understand the types of coping strategies employed by some men, there is a need to further our understanding of variables that influence the choice and utilisation of these strategies. For example, a replication of the study conducted by Matheson and colleagues [33] within a population of men with PCa would be helpful in this regard. As part of developing our understanding further about the role of masculine norms in the process of coping with PCa, an interesting research question might be the extent to which gender role conflict (GRC) might moderate the relationship between PCa variables and positive psychological well-being. For example, does GRC moderate the relationship between approach, problem, and emotion-focused coping strategies and psychological well-being? Finally, in thinking about how we might better support men during the treatment process for PCa, future research should seek to assess medical professionals’ awareness of psychological consequences of medical treatment and how to encourage adaptive self-initiated coping strategies men can employ to mitigate these impacts.

### Study limitations

Limited attention was directed within included studies to considering dynamic aspects of coping, i.e. how the self-initiated coping strategies employed by men in response to PCa might change over the course of the illness. Focusing on characterisation of coping strategies rather than the more dynamic processes of how these might change over time and in response to contextual factors is not limited to studies looking at men’s coping responses to PCa. The more dynamic aspects of coping are rarely specifically addressed within qualitative research investigating male coping more broadly. Regarding the process of PCa diagnosis and treatment, it was not possible within this review to offer insight into how coping might change across the course of the illness (due to the nature of the included studies). Factors relating to disease progression and treatment, however, could be an important contributor in men’s psychological responses and self-initiated coping strategies. This would therefore be deserving of further investigation. Taking this into account, a further limitation of this review is the lack of consideration of how other contextual factors might influence coping strategy selection (e.g. investigating the influence of interpersonal relationships in choice of coping strategies).

In terms of methodological limitations of the current review, the extraction of coping strategies from included papers and the categorisation of these into the suggested meta-themes is a somwhat  subjective process. In part, this is influenced by the variable conceptualisations of coping that were adopted by the included papers (which is an issue often highlighted within the coping literature more broadly). The use of clear inclusion/exclusion criteria and a clear operationalization/definition of coping adopted by this review helped to increase objectivity in this regard. However, the review studies included little in the way of reflexivity around how the world-views and personal characteristics of the researchers may have impacted upon the way in which they engaged with their own data and presented their findings. Again, this hindered the current review when thinking about the wider implications and the applicability of findings.

Finally, it is important to acknowledge the important impact of cohort/age effects that may have influenced these findings. The nature of, and adherence to, masculine norms positioned as ‘traditional’ or hegemonic may change over time. However, there was little focus in the current review around the impact of adherence to masculine norms and how norms might change as a result of contextual factors. Therefore, future reviews/research may like to compare findings between different age groups and cohorts to consider whether this impacts the meta-themes reported here.
